# 757 Diabetes Mellitus Associated with Worse Outcomes in Stevens-Johnson Syndrome and Related Diseases

**DOI:** 10.1093/jbcr/irad045.232

**Published:** 2023-05-15

**Authors:** Keshav Chandran, Deepak Ozhathil

**Affiliations:** Medical University of South Carolina, Charleston, South Carolina; South Carolina Burn Center at MUSC, Charleston, South Carolina

## Abstract

**Introduction:**

Stevens-Johnson Syndrome(SJS),toxic epidermal necrolysis(TEN), and SJS/TEN Overlap Syndrome are on the same spectrum of severe blistering skin diseases affecting mucosal surfaces of the body.They have an immunological etiology and are caused by toxins, drugs, or infectious agents. The mortality rate for patients with these conditions ishigh, but there is limited research on the outcomes for those patients with additional comorbidities.This study investigates the association of inflammatory conditions such as diabetes mellitus, obesity, and tobacco usage have on the outcomes of patients with SJS or related disease. It was predicted that each of these comorbidities would be associated with higher rates of mortality and disease complications.

**Methods:**

This study is a retrospective observational cohort study using the TriNetX Research Network, which provides deidentified electronic medical data from health care organizations all over the world. TriNetX was used to perform a search and analysis for patients with SJS, SJS-TEN Overlap Syndrome, and TEN as well diabetes mellitus(DM), tobacco usage(TU), or overweight/obesity status.A fourth, control cohort of patients without any comorbidities was also included. In total, 8,962 patients were included in the study: 6,348patientsin the control group;426 patients in the TU group; 1,186 patients in the DMgroup, and 1,002 patients in the Obesity group. Outcomes measured were rate and incidence of pneumonia, sepsis, and endotracheal intubation. Outcome variables from each of the four cohorts were analyzed with a total of six unique comparisons. Statistical analyses used included measure of association, Kaplan-Meyer Survival Curve, and measure of incidence.

**Results:**

Compared to the control group, the DM group had a mortality rate14.3% higher(CI 95%, 11.8% -16.9%), pneumonia rate7.6% higher (CI 95%, 5.4%-9.9%), sepsis rate 4.7% higher (CI 95%, 6.9%-11.4%), and endotracheal intubation rate of 1.9% higher (CI 95%, 0.8%-3.0%). Furthermore,compared to the Obesity group, the DM group had a mortality rate 11.6% higher (CI 95%, 8.5%-14.8%)and sepsis rate 4.7% higher (CI 95%, 1.7%-7.6%). Compared to the TU group, the DM group had a mortality rate 12.4%higher(CI 95%, 8.5%-16.3%)and sepsis rate 8.1% higher (CI 95% 4.7%-11.6%). Besides this data on the DM group, the Obesity group showed statistically significant worse outcomes as well. Compared to the control group, the Obesity group showed a mortality rate 2.7% higher (CI 95%, 0.5%-4.9%), pneumonia rate 5% higher (CI 95%, 2.7%-7.3%), sepsis rate 4.5% higher (CI 95%, 2.3%-6.6%), and intubation rate of 2.1% higher (CI 95%, 0.8%-3.4%).

**Conclusions:**

Diabetes mellitus is associated with worse outcomes for patients with SJS, SJS-TEN overlaps, or TEN compared to obesity and tobacco usage.

**Applicability of Research to Practice:**

This data argues the importance of examining a patient’s past medical history when evaluating their prognosis for SJS, SJS-TEN overlaps or TEN.

